# A micro-fabricated device (microICSI) improves porcine blastocyst development and procedural efficiency for both porcine intracytoplasmic sperm injection and human microinjection

**DOI:** 10.1007/s10815-023-03018-0

**Published:** 2024-01-18

**Authors:** Hanna J. McLennan, Shauna L. Heinrich, Megan P. Inge, Samuel J. Wallace, Adam J. Blanch, Llewelyn Hails, John P. O’Connor, Michael B. Waite, Stephen McIlfatrick, Mark B. Nottle, Kylie R. Dunning, David K. Gardner, Jeremy G. Thompson, Allison K. Love

**Affiliations:** 1 Fertilis Pty Ltd, Frome Road, Helen Mayo South, The University of Adelaide, Adelaide, SA 5005 Australia; 2 Virtual Ark Pty Ltd, 73 Woolnough Road, Semaphore, SA 5019 Australia; 3 School of Biomedicine, Faculty of Health and Medical Sciences, The University of Adelaide, Adelaide, SA 5005 Australia; 4 Robinson Research Institute, Adelaide Medical School, The University of Adelaide, Adelaide, SA 5005 Australia; 5 Australian Research Council Centre of Excellence for Nanoscale BioPhotonics, The University of Adelaide, Adelaide, SA 5005 Australia; 6 Institute for Photonics and Advanced Sensing, The University of Adelaide, Adelaide, SA 5005 Australia; 7 Melbourne IVF, East Melbourne, VIC 3002 Australia; 8 School of BioSciences, University of Melbourne, Parkville, VIC 3010 Australia; 9 ART Lab Solutions Pty Ltd, 10 Pulteney Street, Adelaide, SA 5005 Australia

**Keywords:** Intracytoplasmic sperm injection (ICSI), Oocyte, 3D-printing, Embryo, ICSI workflow

## Abstract

**Purpose:**

Intracytoplasmic sperm injection (ICSI) imparts physical stress on the oolemma of the oocyte and remains among the most technically demanding skills to master, with success rates related to experience and expertise. ICSI is also time-consuming and requires workflow management in the laboratory. This study presents a device designed to reduce the pressure on the oocyte during injection and investigates if this improves embryo development in a porcine model. The impact of this device on laboratory workflow was also assessed.

**Methods:**

Porcine oocytes were matured *in vitro* and injected with porcine sperm by conventional ICSI (C-ICSI) or with microICSI, an ICSI dish that supports up to 20 oocytes housed individually in microwells created through microfabrication. Data collected included set-up time, time to align the polar body, time to perform the injection, the number of hand adjustments between controllers, and degree of invagination at injection. Developmental parameters measured included cleavage and day 6 blastocyst rates. Blastocysts were differentially stained to assess cell numbers of the inner cell mass and trophectoderm. A pilot study with human donated MII oocytes injected with beads was also performed.

**Results:**

A significant increase in porcine blastocyst rate for microICSI compared to C-ICSI was observed, while cleavage rates and blastocyst cell numbers were comparable between treatments. Procedural efficiency of microinjection was significantly improved with microICSI compared to C-ICSI in both species.

**Conclusion:**

The microICSI device demonstrated significant developmental and procedural benefits for porcine ICSI. A pilot study suggests human ICSI should benefit equally.

**Supplementary Information:**

The online version contains supplementary material available at 10.1007/s10815-023-03018-0.

## Introduction

Intracytoplasmic sperm injection (ICSI) has significantly expanded the accessibility of assisted reproductive technologies (ART) to patients with male factor infertility, with demand predicted to increase [1]. With the first human pregnancies reported in 1992 [2], ICSI is now performed in ~67% of IVF cycles globally, with differences in its use between countries ranging from 30–100% of IVF cycles [3]. ICSI is one of the most technically difficult procedures conducted by embryologists with experience and expertise key factors in obtaining optimal results [4]. As a result, there is an increased procedural burden for clinics and expert availability is limited [5, 6]. There is also a training burden, with staff needing to achieve and maintain strict performance benchmarks to perform ICSI [7]. Several parameters have been reported to impact outcome including the number of injections performed by personnel, polar body orientation relative to the injection pipette, time from oocyte collection to ICSI, and insertion and depth of injection pipette penetration [4, 8–11].

ICSI is dependent on micromanipulation equipment, and the fundamentals of an ICSI workstation have changed little since first described [2]. A typical ICSI set-up consists of an inverted microscope with a contrast optical system equipped with 10 to 40 times objectives, two micromanipulators, one for moving the holding pipette and another for the injection pipette, an oil or air-pressure regulated microinjector for holding the oocyte with a heat-polished glass holding pipette during injection, and an air or oil-hydraulic controlled microinjector. Advances in equipment utilised for ICSI to either ease the embryologist’s technical capacity and time of procedure are limited [12–20], with new holding pipette designs, Piezo ICSI, and robotic ICSI only adding further complexity.

In comparison to modifications of the injection procedure and equipment, little research has been performed on how oocytes are restrained for the ICSI procedure. To date, the heat polished holding pipette has been the “gold-standard” to perform this function and relies on negative suction pressure on the oocyte to ensure correct positioning. However, it is the design constraints of the pipette, coupled with the design of conventional ICSI pipettes, that causes oolemma invagination [21]. Furthermore, there is the potential for cytoplasmic stress from the act of suction and the accompanying need to withdraw cytoplasm into the injection pipette to ensure the oolemma is pierced [22], which is overcome by piezo injection [13, 23, 24].

The present study builds on previous published work that demonstrated the utility of two-photon polymerization (2PP) 3D-printing technology to create a two-part microscale oocyte holding device that improved the traceability and the efficiency of mouse oocyte injection [25]. This approach has been enhanced by creating a single piece printed ICSI support device to hold up to 20 oocytes at a time, a number chosen through consultation with clinical embryologists. This device is referred to here as microICSI. The functional and work-flow differences between using microICSI and conventional ICSI (C-ICSI) were compared, assessing both procedural efficiency and porcine embryo developmental outcomes. Finally, a pilot study demonstrates the utility of microICSI for human oocyte microinjection.

## Materials and methods

Unless otherwise specified, all chemicals and reagents were purchased from Merck-Sigma-Aldrich (St. Louis, MO, USA) and sterile polystyrene cell culture consumables were sourced from Falcon by Corning (New York City, NY, USA).

### Fabrication of microICSI

**Fig. 1:**
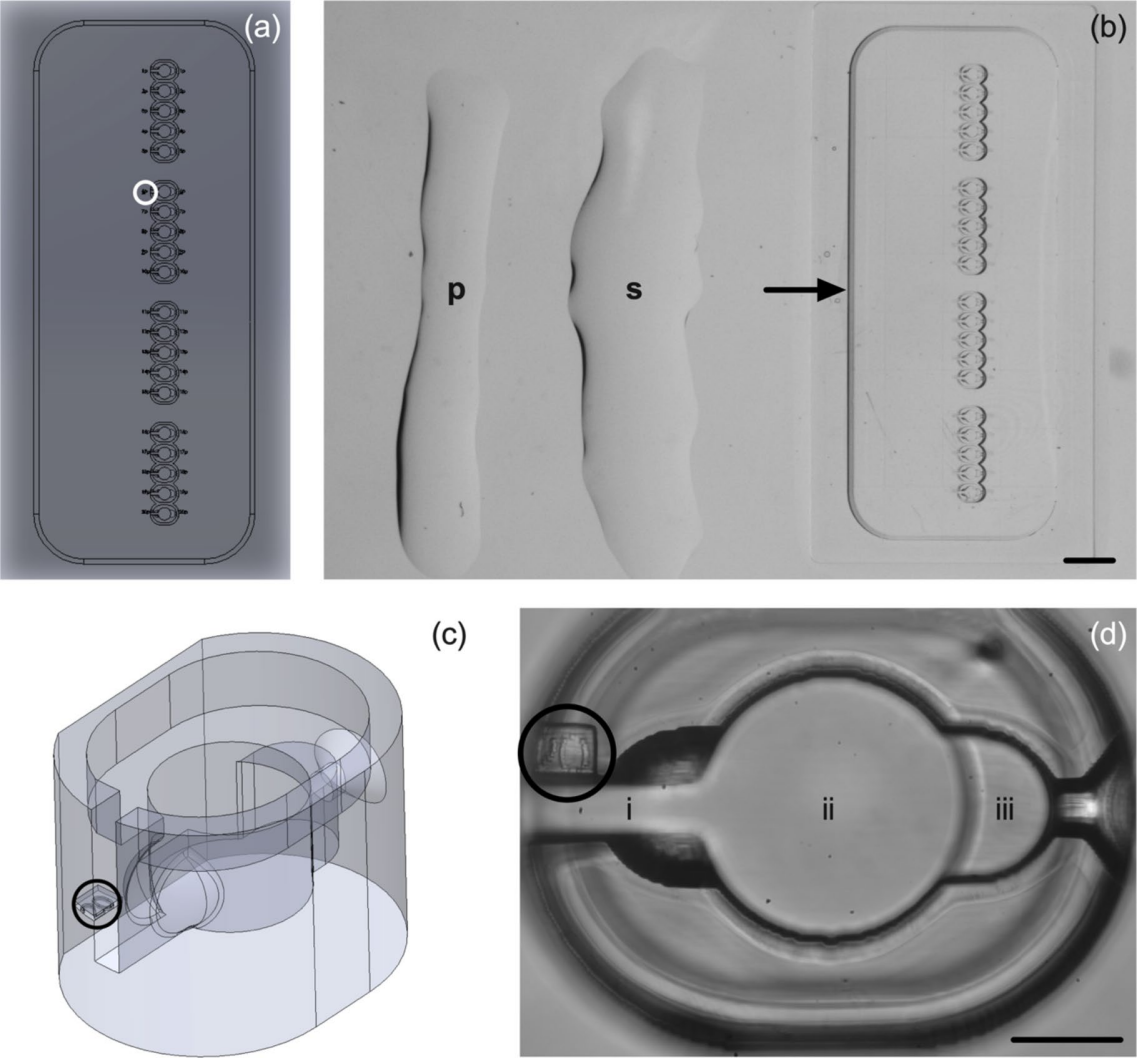
The microICSI array (scale bars (b) = 1 mm, (d) = 100 μm); **a** the computer-aided design (CAD) drawing of the microICSI array to support up to 20 oocytes in individual microwells, with the circle indicating the location of a ‘base number’; **b** the 2PP printed array in an ICSI dish filled with media under oil, where ‘**s**’ indicates the elongated 3.5% PVP drop used for sperm incubation, allowing workflow to be directly horizontal to each well, ‘**p**’ indicates the strip of 7% PVP, and the arrow points to the boundary of the ‘moat’ that contains the medium; **c** oblique angle of a single microICSI well to show the 3D features, with the circle indicating the ‘focal plane number’; **d** a single microwell filled with media under oil, (i) injection pipette slot, (ii) microwell, and (iii) injection hemisphere, circle = focal plane number

The microICSI array was designed to support 20 oocytes during the ICSI process within a hemisphere structure to remove the need for a holding pipette and suction (Fig. 1 a and b). The oocyte is cupped during the injection within the hemisphere surrounded by a microwell for improved oocyte traceability and systematic workflow (Fig. 1 c and d). A slot allows the injection pipette to manoeuvre oocytes into position within the hemisphere prior to sperm pickup, including polar body orientation (Fig. 1d). The array includes numbers printed into the base that are visible under a dissecting microscope for oocyte handling (Fig. 1a). The numbering of wells is further repeated within the focal plane of the midline of the oocyte visible under an ICSI inverted microscope (Fig. 1c).

The microICSI arrays were printed with a NanoOne 1000 high-resolution 2PP 3D printer and a proprietary acrylate-based 2PP resin (‘UpFlow’, UpNano GmbH, Vienna, Austria). Following modelling of 3D parts in CAD software (‘Solidworks’, Dassault Systѐmes Solidworks Corp., Waltham, MA, USA), exported STL files were imported into the Think3D software (version 1.7.3, UpNano GmbH). Parts were printed using a 10× objective operating in coarse mode using ‘voxel slicing’, with a layer thickness of 5 μm, line distance of 4 μm, and power setting of 430 mW.

The microICSI arrays were post processed by washing in baths of propylene glycol monomethyl ether acetate (PGMEA ≥99.5%) for 3 × 10 min and 2-propanol (99.9%) for 1 × 3 min and 1 × 2 min before removal from the print substrate with a razorblade and subsequent air drying. Dried parts were cured for 5 min in an ODS Cure Box (One Digital System, Incheon, Korea) at 60% power before plasma treatment. microICSI arrays were treated with an oxygen plasma at a pressure of 780–820 mTorr for 60 s using a PDC-002-HP chamber (Harrick Plasma, NY, USA). Plasma treated arrays were adhered to commercial polystyrene dishes (Product N^o^: 16006, Vitrolife Pty. Ltd., Sydney, Australia) using a small amount (~25–30 mg) of UpFlow resin followed by 2 × 5 min curing treatments in the ODS Cure Box.

After curing, dishes were washed twice in 3 mL of 96% ethanol (Chem-Supply Pty Ltd, Gillman, SA, Australia) and rinsed twice in filtered (pore size 0.22 μm; Millipore, Bedford, MA, USA) 5% 7X detergent (MP Biomedicals Australasia, Seven Hills, NSW, Australia). Dishes were then rinsed three times in Milli-Q water to remove residual detergent and left to dry on a heat plate set to 37.5 °C overnight (Ratek Instruments Pty Ltd, VIC, Australia). At each step of washing, a P1000 micropipette was used to pipette washing solution over the microICSI array, thereby flushing the wells.

For human oocytes, an adjustment to the size of the hemispheres was performed to accommodate the larger size of oocytes prior to printing the microICSI arrays. Thereafter, printing, post-processing and washing were conducted as described above.

Toxicity testing of previous 2PP parts were shown to be non-toxic [25–27] using a mouse embryo assay (MEA) that included both negative and positive controls (IVF VET Solutions, SA, Australia). This was repeated in the current study with no toxicity detected. All subsequent printed arrays used for experimentation followed the same washing protocol detailed above.

### Oocyte collection and maturation

Unstimulated porcine ovaries collected from a local abattoir from cycling gilts and sows were transported back to the laboratory in phosphate buffered saline (PBS) in a thermos between 30 and 35 °C. Ovaries were washed in warm saline and placed into beakers within a water bath set to 37 °C. Clear antral follicles between 3 and 6 mm were aspirated with a 21-gauge needle with constant suction (1 L/min) into 9 mL Greiner No Additive Vacuette tubes (Interpath Services, Somerton, VIC, Australia). Cumulus-oocyte complexes (COCs) with several layers of cumulus cells were isolated from the aspirant. Once pooled together, COCs were washed 3 times and placed into groups of 50 in 500 μL of *in vitro* maturation (IVM) medium consisting of Medium 199 (Gibco, Thermo Fisher Scientific, Waltham, MA, USA) supplemented with 10 IU/mL equine chorionic gonadotropin (Folligon MSD, Australia), 10 IU/mL human chorionic gonadotropin (hCG, Chorulon MSD, Australia), 5 μg/mL insulin, 10 ng/mL epidermal growth factor (EGF), 1 mM cysteamine, 100 μg/ml Na-pyruvate, 75 μg/mL Penicillin-G, 50 μg/mL Streptomycin sulphate, and 10% filtered sow follicular fluid under mineral oil (Origio, Sydney, NSW, Australia) in a 4-well NUNC dish (Thermo Fisher Scientific). Oocytes were matured for approximately 40 h in a humidified atmosphere of 5% CO_2_ in air at 38.5 °C.

At the end of incubation, morphologically mature COCs were isolated using a small-bore glass pipette (Rowe Scientific, Lonsdale, SA, Australia). Groups of 50–60 COCs were placed into separate 5 mL round bottom tubes with IVM medium without any hormones for transportation to the ICSI laboratory at 37 °C in a transport incubator (CryoLogic, Blackburn, VIC, Australia) until time of denudation, approximately 1 h after cessation of maturation.

### Intracytoplasmic sperm injection (ICSI) and microICSI

For C-ICSI, two 5 μL drops of 7% polyvinylpyrrolidone (PVP; Origio Australasia Pty Ltd, CooperSurgical, Denmark) for priming the injection pipette and seven drops of 5 μL of 4-(2-hydroxyethyl)-1-piperazineethanesulfonic acid (HEPES) buffered NCSU23 (ART Lab Solutions Pty Ltd, Adelaide, SA, Australia) were placed into a Vitrolife ICSI dish in a 3 × 3 droplet grid (Online Resource 1). Five microlitres of HEPES-buffered NCSU23 were added to the central PVP drop to make a 3.5% PVP solution for sperm to partially slow motility as it was observed 7% PVP abruptly stopped motility of porcine sperm. The dish containing the microICSI array differed in medium arrangement, comprising two strips of 5 μL of 7% PVP, laid down the left side of the microICSI array, with 5 μL of HEPES-buffered NCSU23 added to the PVP droplet closest to the array for sperm pick up (Fig. 1b). Twenty microlitres of HEPES-buffered NCSU23 was pipetted over and around the microICSI array moat (Fig. 1b). All ICSI dishes were covered with 5 mL of MEA tested paraffin oil (Merck Group, Macquarie Park, NSW, Australia; Online Resource 1) and heat equilibrated to 38.5 °C for a minimum of 3 h.

Freshly collected and extended semen was purchased from a local boar stud (Sabor, Clare, SA, Australia). Semen was equilibrated to room temperature (~24 °C) from storage temperature (18 °C) and was centrifuged at 300 g for 5 min. The supernatant was discarded, and the remaining pellet was resuspended in 10 mL of warm sperm wash media (Medium 199 supplemented with 0.1 mg/mL sodium pyruvate, 0.9 mg/mL calcium-lactate, 0.075 mg/mL penicillin G, 0.05 mg/mL streptomycin sulphate, and 10% fetal bovine serum (FBS)). The resuspended pellet was then centrifuged as previously described and further resuspended in sperm wash medium. Sperm were further diluted with warm HEPES-buffered NCSU23 to obtain a final concentration of ~5 × 10^6^ sperm/mL.

Mature COCs were treated with 0.1 mg/mL hyaluronidase in HEPES-buffered NCSU23 for one minute and gently pipetted using a 130- to 140-μm internal diameter (ID) Flexipet pipette tip and Cook Adjustable Handle (Cook Medical Pty Ltd, Eight Mile Plains, QLD, Australia) to remove cumulus cells. Oocytes were washed three times in HEPES-buffered NCSU23 post denuding in a four-well NUNC dish without oil overlay and assessed for oocyte membrane integrity and the presence of a single polar body for selection to undergo the ICSI procedure. Due to variation in follicle size and numbers on each ovary, treatment group sizes varied from 10 to 20 oocytes. Analysis of data was standardized to a set number of oocytes, as described below.

Pipette setup was timed for the C-ICSI and microICSI procedure, as only one injection pipette is required for microICSI as opposed to a holding pipette and injection pipette for C-ICSI. This included the time taken for focussing on the medium drop and pipettes during pipette positioning in media under oil for both treatments. Two ICSI workstations were utilized to allow operators to conduct experimentation concurrently and avoid oocyte aging. A Nikon ICSI station utilized a Nikon TE2000 Eclipse inverted microscope (Nikon Corporation, Tokyo, Japan) combined with Eppendorf micromanipulators (Eppendorf, Hamburg, Germany) and air microinjector (Eppendorf) for the 25 μm ID holding pipette and oil microinjector (Eppendorf) for the 6-μm ID injection pipette, both sourced from ICSION (Thebarton, SA, Australia). Both pipettes had an angle of 30°. A second Olympus station comprised an Olympus IX83 inverted microscope (Evident Corporation, Tokyo, Japan) and Narishige ON4 micromanipulators (Tokyo, Japan), with Narishige injectors (IN-21 for oil: injector, IN-9B for air: holding pipette). Treatment order per operator was randomized for each replicate. Operators used a combination of 10 × objective with 2 × magnifier on the Olympus microscope and 20 × objective on the Nikon microscope to visualise sperm and perform the ICSI process.

For C-ICSI, within a custom-built modified heated infant crib set to 38.5 °C, 10–20 matured oocytes were transferred into the 5-μL HEPES media droplets using a 170-μm flexi-pipette just prior to the ICSI procedure under a Nikon SMZ745T dissecting microscope (Nikon Corporation). Approximately 3 μL of diluted sperm was pipetted into the 3.5% PVP droplet using a flexi-pipette. The dish was carefully transferred to either inverted microscope onto a heated stage set to 38.5 °C. For each injection, a single motile sperm with good morphology was selected and immobilized within the 3.5% PVP sperm droplet. An oocyte was held in place with the holding pipette, with the polar body rotated to either the 6 or 12 o’clock position using the injection pipette. The injection pipette with the sperm located at the tip was introduced into the oocyte at the 9 o’clock position, with the ooplasm aspirated into it to ensure the oolemma was ruptured. Time of injection was recorded from when the oocyte was in focus and the holding pipette entered the drop to when the oocyte was released from the holding pipette. Invagination was scored in reviewed video footage based on previous literature [28], with 1 being the lowest and 3 being the highest, by estimating the level of oolemma invagination when injection pipette enters the oocyte at the 9 o’clock position for both treatments. The number of hand adjustments between controllers required for each single oocyte injection was quantified by video recordings (Online Resource 2-4). An adjustment was defined as an operator’s hand leaving one controller and then using another separate controller.

For microICSI, 10–20 matured oocytes were transferred into the microICSI microwells containing 20 μL of HEPES media within the moat with a 170-μm ID flexi-pipette on the same heated dissecting microscope used above. The 170-μm tip fits within the top of the microwell to aid oocyte loading and unloading (Fig. 1c). All oocytes were manoeuvred into the injection hemisphere within each microwell using the injection pipette and orientated to allow the polar body to be at either 6 or 12 o’clock. The time taken to orient 10 oocytes was recorded and averaged on a per oocyte basis. Sperm were loaded into the injector as per C-ICSI above; the injector entered the microwell through the injection slot; the injection was performed on the correctly orientated oocyte with the ooplasm aspirated into it to ensure the oolemma was ruptured. The injection pipette was drawn back through the slot, gently easing the pipette from the oocyte before gently pushing the oocyte back to the middle of the well with the pipette. The amount of time taken for injection during microICSI was recorded from when the oocyte and the injection pipette within the pipette slot were in focus until when the injection pipette was removed from the oocyte. Oocytes were excluded from culture if injection was unsuccessful for both C-ICSI or microICSI.

Sham injections were conducted using microICSI to assess parthenogenic activation and subsequent embryo development. For sham injections, oocytes were injected with 7% PVP and no sperm. Non-injected oocytes were also placed separately into culture as controls. Both shams and controls act as developmental controls and are only presented in graphs comparing developmental rates. All other data compares C-ICSI to microICSI. All injected oocytes within treatment groups were washed three times in a 35-mm culture dish heated to 38.5 °C containing 2.5 mL NCSU23 before incubation.

### Embryo culture

Presumptive zygotes, sham injected, and controls were cultured in groups of 5 in 50 μL drops of modified NCSU-23 within a 60-mm petri dish (Falcon Product N^o.^: 351007) covered with ~8 mL of MEA-tested paraffin oil until day 4 in a humidified incubator at 38.5 °C (6% CO_2_, 7%O_2_, balanced with N_2_). At day 4 of culture, cleavage was scored, as well as post-injection survival rate, defined here as maintaining membrane integrity, and the degree of fragmentation in both un-cleaved and cleaved embryos. Fragmentation was defined as an embryo with greater than 30% of the perivitelline space and space between cells occupied by small, membrane-bound vesicular bodies. All surviving embryos were moved to equilibrated dishes with modified blastocyst NCSU-23 (ART Lab Solutions Pty Ltd), supplemented with 10% FBS and overlayed with paraffin oil. Blastocysts were scored on day 6 of culture.

#### Differential staining

The protocol for differential staining was based on a study by Grupen et al. with some minor modifications as outlined below [29]. Blastocysts produced from four replicates obtained from both techniques were collected for staining immediately after blastocyst scoring on day 6. Blastocysts were washed in warmed (37 °C) HEPES-buffered NCSU23 (ART Lab Solutions Pty Ltd) for differential staining. The zona pellucidae were removed by incubation with 0.5% pronase dissolved in PBS for approximately 2 min, then washed in HEPES-MEM-PVA (Minimum Essential Medium (Medium 199, HEPES (Gibco, Thermo Fisher Scientific) and 1 mg/mL polyvinyl alcohol (PVA)) and then further incubation in 10 mM trinitrobenzenesulphonic acid (TNBS) in HEPES-MEM-PVA on ice for 15 min. Blastocysts were then removed and further washed in HEPES-MEM-PVA prior to being incubated with 0.2 mg/mL anti-dinitrophenol BSA in HEPES-MEM-PVA at 38°C for 10 min. Blastocysts were then washed again in HEPES-MEM-PVA and incubated in HEPES-MEM-PVA containing ~10% guinea pig complement serum, 0.01 mg/mL propidium iodide, and 0.05 mM bisbenzimide (Hoechst 33258) for 10 min. After washing, blastocysts were transferred into HEPES-MEM-PVA as a post incubation step, then were dehydrated in 100% ethanol (Chem-Supply Pty Ltd, Gillman, SA, Australia) and mounted on a microscope slide in 2 μL of glycerol underneath a cover slip. Fluorescence images of the nuclei of blastocysts were captured with an epifluorescence inverted microscope at 20× objective (Eclipse TS100; Nikon Corporation; excitation filter 330–380 nm and barrier filter 420 nm). Numbers of blue inner cell mass (ICM) and pink to red trophectoderm (TD) cells were manually counted post imaging by one researcher blinded to treatments using the Fiji Is Just ImageJ (FIJI) freeware [30].

### Human microICSI validation

Vitrified human oocytes (*n* = 18, from two patients) consented to research (The University of Adelaide Ethics Approval Number H-2023-082) were obtained from a local IVF clinic and warmed in the laboratory according to the SAGE vitrification warming kit instructions (Origio Australasia Pty Ltd). After warming, oocytes were washed in GMOPS PLUS (Vitrolife Pty Ltd) and placed in an incubator at 37 °C for at least 1 h before the ICSI procedure.

For C-ICSI, one 5 μL microdrop of 7% PVP (Origio Australasia Pty Ltd) was placed in the centre of a Vitrolife ICSI dish surrounded by eight drops 5 μL of GMOPS PLUS. The microICSI dish comprised of one 5 μL strip of 7% PVP laid down the left side of the microICSI array, with 20 μL of GMOPS PLUS pipetted over and around the microICSI array moat. All ICSI dishes were overlayered with 5 mL of paraffin oil (Merck Group) and equilibrated to 37 °C for a minimum of 3 h.

Oocytes were loaded into the C-ICSI dish or microICSI array using a 170-μm Flexipet pipette tip as described for pig oocytes. Instead of sperm, 4 μm TetraSpeck™ microspheres (Thermo Fisher Scientific) that were centrifuged and washed in GMOPS PLUS medium were loaded into the 7% PVP droplets in each dish as a visual substitute for spermatozoon injection into human oocytes. A single bead was aspirated into the 5-μm ID injection pipette (Origio Australiasia Pty Ltd) from the PVP droplet. The injection process followed as per porcine C-ICSI and microICSI. The time for the bead injection and the number of hand adjustments were recorded as for porcine ICSI. Following successful injection, oocytes were observed for lysis after 90 min of culture. If un-lysed, they were reinjected to provide further timing and hand adjustment data. Immediately after the re-injection data was obtained, oocytes were discarded as biohazard material.

### Statistical analysis

All statistical analysis was conducted using GraphPad Prism v9.5.1. All data were tested for normality to determine whether parametric or non-parametric analyses were applied. All binomial data was arcsin-transformed to make the data continuous for statistical comparison but is graphed as binomial data. Where appropriate, paired comparisons were used to account for replicate effects. The statistical test used, replicate numbers, sample sizes, and *p*-values are reported in each figure legend respectively. Data are presented as mean ± SEM, and statistical significance was accepted at *P* < 0.05.

## Results

### Effects on preparation time

**Fig. 2:**
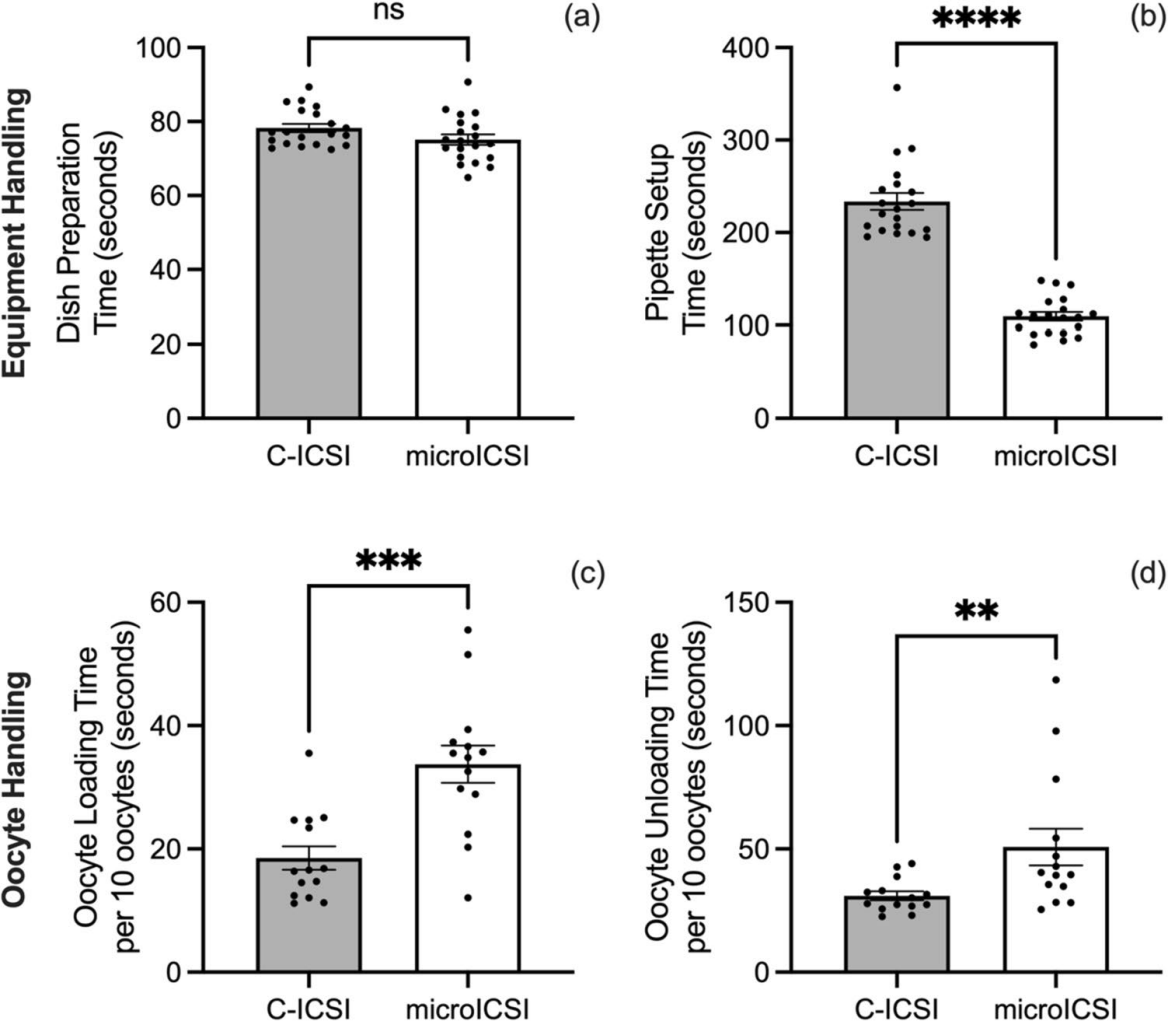
Setup efficiency compared between C-ICSI and microICSI (mean ± SEM); **a** mean time taken to prepare the ICSI dish with media and oil overlay (20 replicates, *n* = 2 dishes prepared per treatment, Mann-Whitney test, *P* > 0.05); **b** mean time taken to setup pipettes for ICSI, including focusing and position setting (20 replicates, *n* = 2 injector setup times on two microscopes, Wilcoxon test, *P* < 0.0001); **c** mean time to load 10 oocytes into an ICSI dish for microinjection (14 replicates, *n* = 4 × 10 oocytes loaded per replicate, Mann-Whitney test, *P* < 0.001); **d** mean time to unload 10 oocytes into an ICSI dish after microinjection (14 replicates, *n* = 4 × 10 oocytes per replicate, Mann-Whitney test, *P* < 0.01)

Dish preparation time for C-ICSI and microICSI was equivalent (Fig. 2a). However, pipette setup time was reduced by half by removing the holding pipette (Fig. 2b). Loading the microICSI microwells added to oocyte handling time for both loading and unloading oocytes by 15–20 s (Fig. 2 c and d).

### Effects on the ICSI procedure

**Fig. 3:**
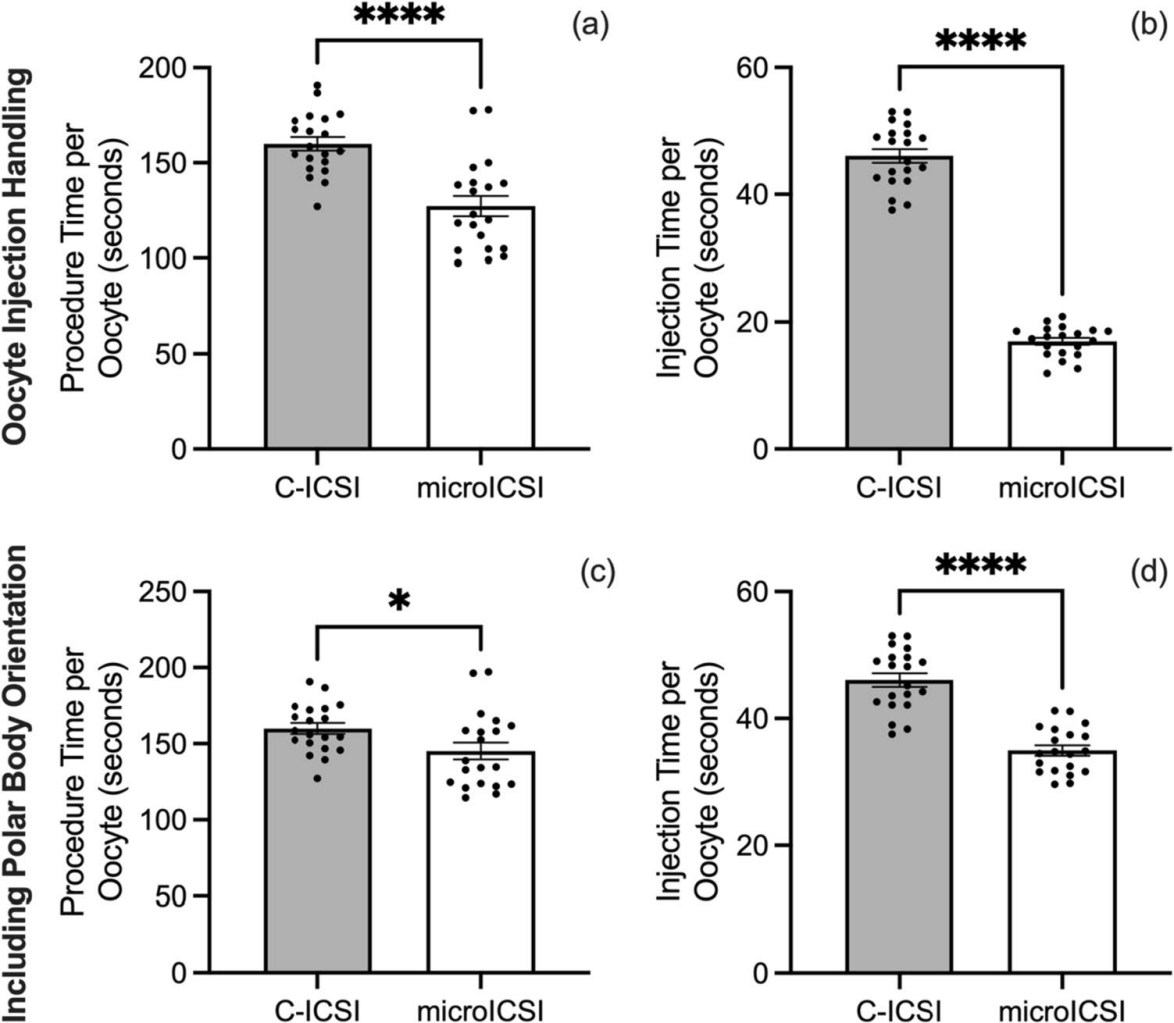
Procedural efficiency compared between C-ICSI and microICSI (mean ± SEM); **a** mean procedure time per oocyte (20 replicates, *n* = 10-20 oocytes, paired *t* test, *P* < 0.0001); **b** mean injection time per oocyte (20 replicates, *n* = 10–20 oocytes, paired *t* test, *P* < 0.0001); **c** mean procedure time per oocyte including microICSI polar body orientation time (20 replicates, *n* = 10–20 oocytes, Wilcoxon test, *P* < 0.05); **d** mean injection time per oocyte including microICSI polar body orientation time (20 replicates, *n* = 10–20 oocytes, paired *t* test, *P* < 0.0001)

**Fig. 4:**
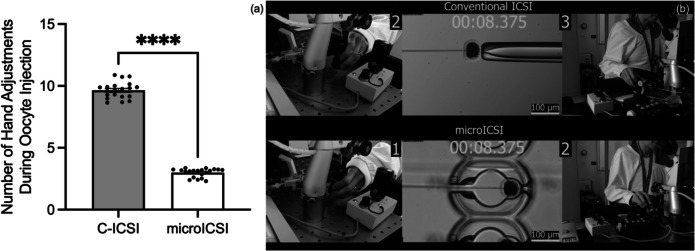
Number of hand adjustments required to perform C-ICSI compared to microICSI (mean ± SEM); **a** mean number of hand adjustments required to conduct ICSI for a single oocyte (20 replicates, *n* = 10–20 oocytes, Mann-Whitney test, *P* < 0.0001); **b** screenshot of Online Resource 2, indicating how hand adjustments were tracked by camera during ICSI

**Fig. 5:**
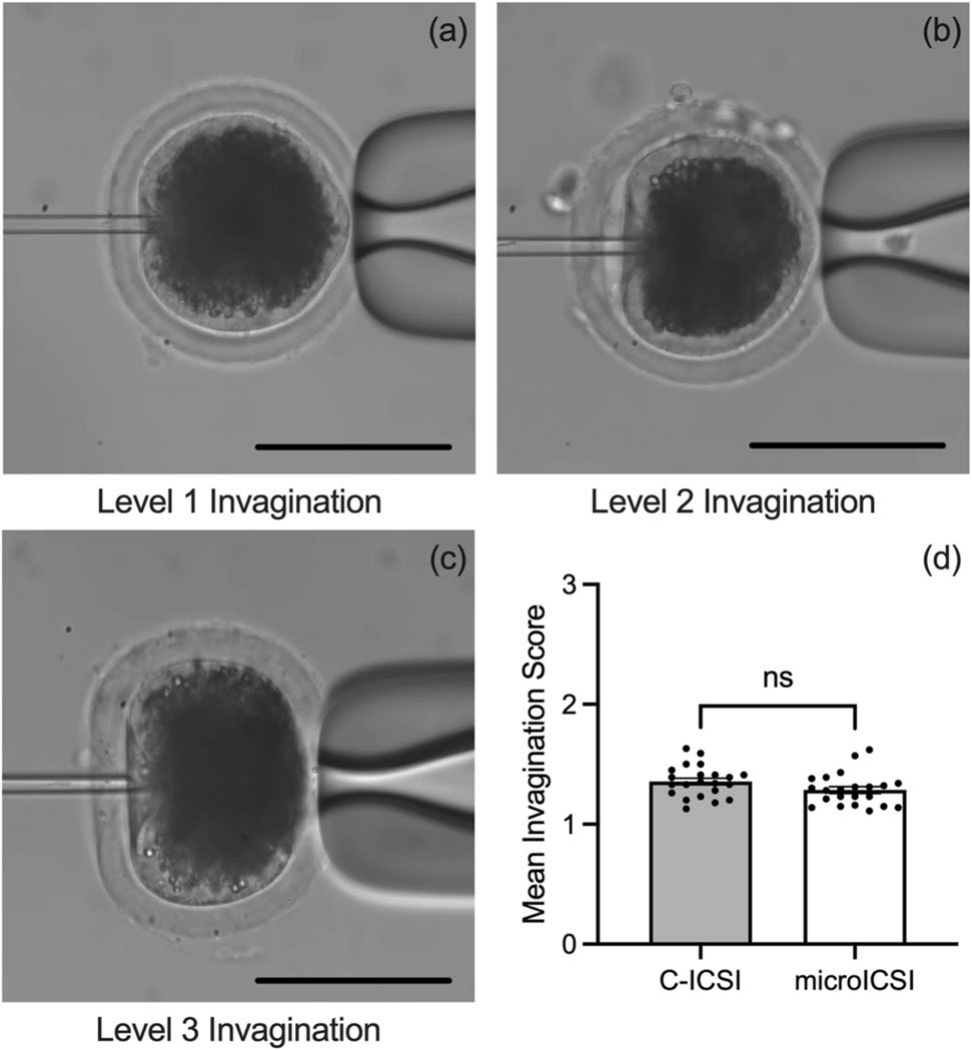
Invagination of the oolemma (mean ± SEM, scale bars = 100 μm); **a** example of a level 1 oolemma invagination; **b** example of a level 2 oolemma invagination; **c** example of a level 3 oolemma invagination; **d** mean invagination of the oolemma (20 replicates, *n* = 10–20 oocytes, paired *t* test, *P* > 0.05)

The average procedural time spent manoeuvring and injecting each oocyte was reduced for microICSI compared to C-ICSI (Fig. 3a). For the injection process, the time was more than halved for each oocyte (Fig. 3b), and a reduction of time was still evident for microICSI even when average polar body orientation time per oocyte was considered (Fig. 3 c and d). Polar body orientation time was equivalent between C-ICSI and microICSI (Online Resource 5). Because the holding pipette was removed with microICSI, the hand adjustments required to perform ICSI were reduced by two thirds, as tracked by the number of hand adjustments between different controllers (Online Resource 2 and 3; Fig. 4). The difference in injection time and hand adjustments between operator and rig was less than 2 s and a single left-hand adjustment respectively (Online Resource 6). The level of invagination was equivalent between C-ICSI and microICSI (Fig. 5). Mean injection failure rate was also equivalent at 2.5% for C-ICSI and 2.7% for microICSI.

### Effect on developmental rate

**Fig. 6:**
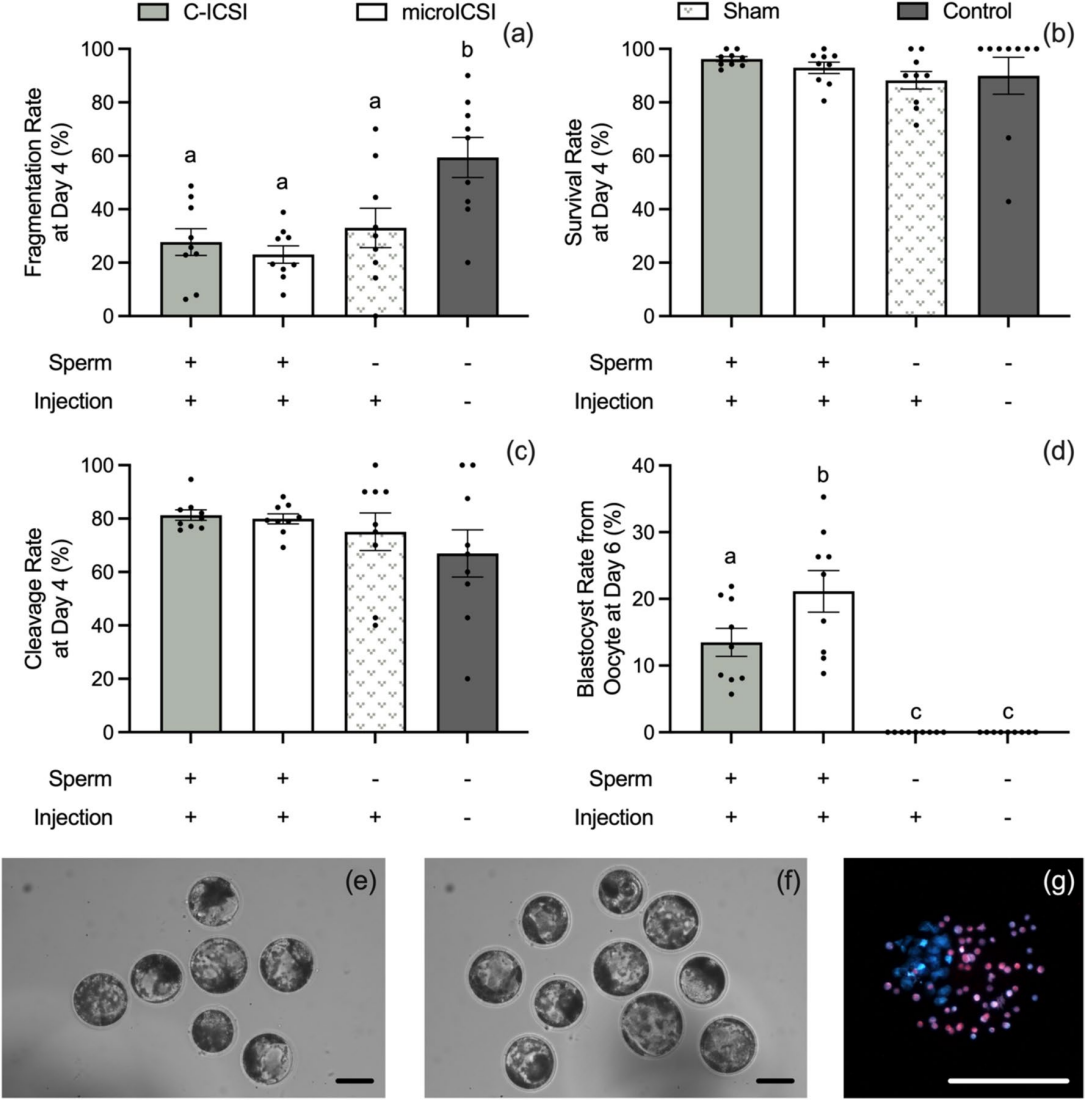
Embryo developmental rates compared between C-ICSI and microICSI (mean ± SEM. Different superscripts indicate significant differences, scale bars = 100 μm); **a** fragmentation rate on day 4 (nine replicates, *n* = 10–20 oocytes, RM one-way ANOVA with Geisser-Greenhouse correction and Tukey’s multiple comparisons test on arcsin, *P* < 0.0001); **b** survival rate at day 4 (nine replicates, *n* = 10–20 oocytes, Friedman test and Dunn’s multiple comparisons test on arcsin, *P* > 0.05); **c** cleavage rate at day 4 (nine replicates, *n* = 10–20 oocytes, Friedman test and Dunn’s multiple comparisons test on arcsin, *P* > 0.05); **d** blastocyst rate at day 6 (nine replicates, *n* = 10-20 oocytes, paired *t* test on arcsin comparing C-ICSI to microICSI, *P* < 0.05); **e** a representative image of C-ICSI porcine blastocysts from a single run; **f** a representative image of microICSI porcine blastocysts from a single run; **g** a representative image of a differentially stained porcine blastocyst

Rates of embryo development remained consistent between treatments, with fragmentation rate, survival rate, and cleavage rates being equivalent across both ICSI techniques (Fig. 6a–c). However, there was a significant increase in blastocyst rate for the microICSI group compared to C-ICSI (Fig. 6d). Cell numbers of ICM and TD were equivalent between the blastocysts produced by C-ICSI and microICSI (Table 1).

**Table 1:** Blastocyst cell numbers compared between C-ICSI and microICSI (four reps, mean ± SEM; matching superscripts indicate *P* > 0.05); sample size (*n*) was higher in microICSI due to the higher blastocyst rate, trophectoderm (TE) cell numbers (Welch’s *t* test, *P* > 0.05), inner cell mass (ICM) cell numbers (Mann-Whitney test, *P* > 0.05), total cell number (TCN; Welch’s *t* test, *P* > 0.05), and percentage of ICM/TCN (Mann-Whitney test, *P* > 0.05)

Treatment	*n*	No. of cells			ICM/TCN (%)
		TD	ICM	TCN	
C-ICSI	16	27 ± 2^a^	7 ± 1^b^	34 ± 3^c^	19 ± 2.5^d^
microICSI	30	33 ± 3^a^	6 ± 1^b^	39 ± 4^c^	16 ± 1.8^d^

### Validation of translation to human oocytes

**Fig. 7:**
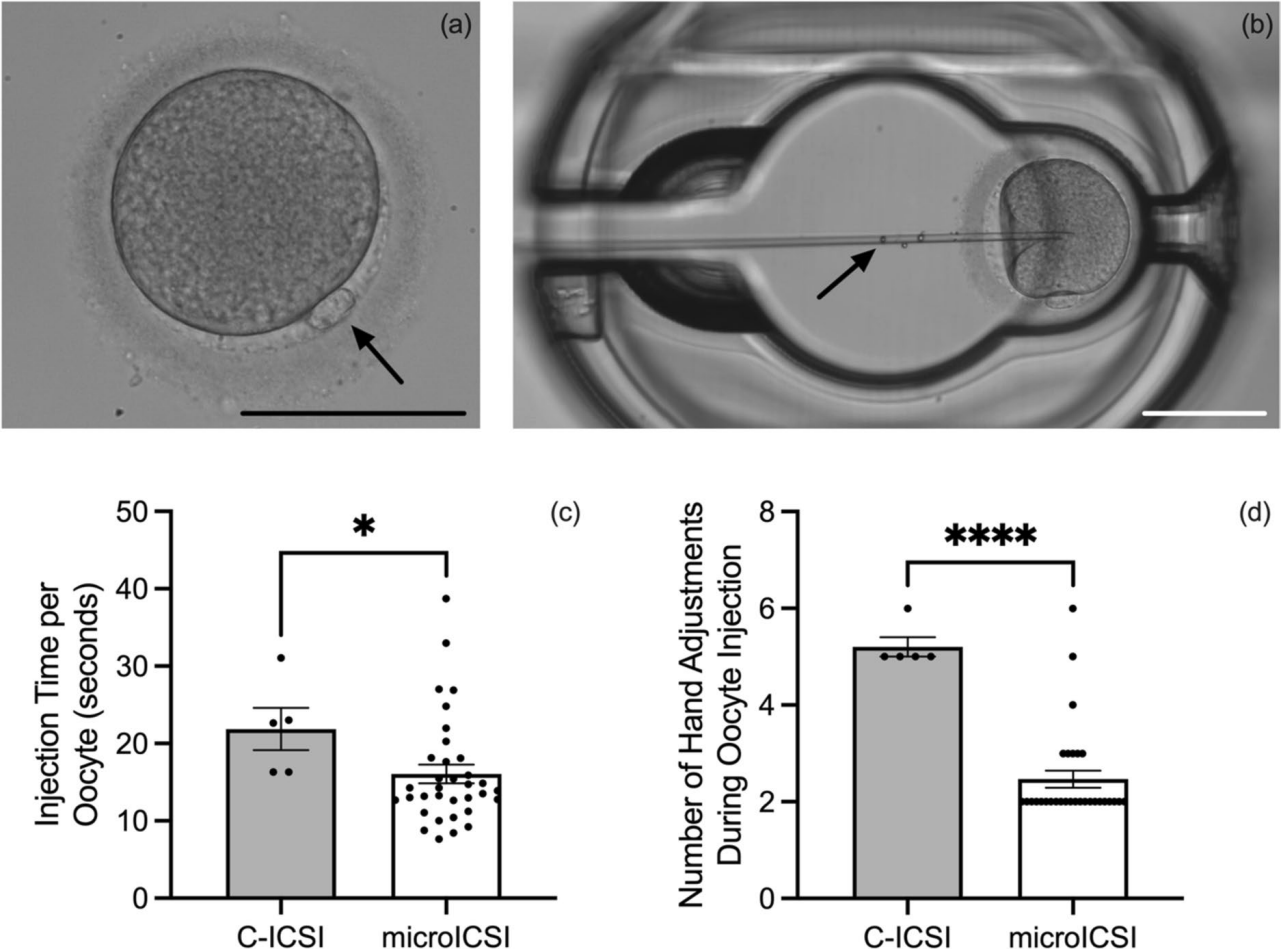
Pilot study of microICSI with human oocytes (mean ± SEM, Scale bars = 100 μm); **a** representative image of a metaphase II stage human oocyte with the arrow pointing to the extruded polar body; **b** representative image of a human oocyte during microICSI injection with the arrow pointing to the bead used to indicate successful sham injection; **c** injection time per individual human oocyte (two replicates, *n* = 5 injections for C-ICSI, 35 injections for microICSI, Mann-Whitney test, *P* < 0.05); **d** number of hand adjustments required to conduct ICSI for individual oocytes (two replicates, *n* = 5 injections for C-ICSI, 35 injections for microICSI), Mann-Whitney test, *P* < 0.0001)

The microICSI device was equally suited to perform microinjection on human oocytes after adjusting the hemisphere size (Online Resource 4; Fig. 7). No immediate lysis was observed following the first round of bead injections and, from 18 oocytes, one oocyte was lysed after 90 minutes and excluded from re-injections. Consistent with porcine results (Figs. 3 and 4), injection time, and hand adjustments were reduced in microICSI compared to C-ICSI (Fig. 7 c and d).

## Discussion

ICSI has transformed the treatment of male infertility by providing access to treatment that maximises the potential fertilization rate for most couples. Nevertheless, ICSI remains one of the most difficult skills to master by embryologists [4]. Furthermore, as an adjunct technique to IVF, it has a significant impact on laboratory workflow and resourcing, which is rarely assessed when examining the impact of adjunct technologies brought into the laboratory [5, 6].

Performing ICSI with the microICSI device delivered a 56% improvement in the blastocyst rate of injected porcine oocytes from 13.5% ± 2% for C-ICSI to 21.1% ± 3% (Fig. 6d–f), with the C-ICSI group matching rates reported in previous research [31]. If such a result translates to human ICSI, then the opportunity for couples to have a live birth from a single ICSI cycle will be significantly enhanced, as approximately one additional embryo should be available. Interestingly, the proportion of oocytes that cleaved, taken as the fertilization rate, did not increase (Fig. 6c). In human oocytes, Piezo ICSI improves the fertilization rate, leading to one more transferable embryo compared to C-ICSI [18, 20]. Piezo ICSI reduces stress on the oocyte cytoplasm by removing the need to withdraw cytoplasm into the pipette [13, 23], a proposed benefit also associated with a borosilicate glass ‘funnel-shaped’ substitute for the holding pipette [16]. The benefit of microICSI could be through the same mechanism, or through the removal of negative pressure suction from the holding pipette. Alternatively, the improved porcine blastocyst rate in microICSI could be a result of reducing the time the oocytes spend out of the incubator, as the procedure was significantly faster than C-ICSI. A future animal model study with equivalent times spent out of the incubator for both groups would determine the embryological outcomes attributable to the change in injection method.

A reduction in blastocyst quality has been observed if the invagination ‘funnel’ persists after injection in human oocytes [32, 33]. Counter to this, the degree of invagination of the oolemma during microinjection has also been reported to reflect the integrity of the membrane, with high levels of invagination correlating to embryo survival, cleavage, and quality [21, 34]. While invagination may be a strong indicator of oocyte membrane integrity, invagination likely places pressure on the cytoplasm and organelles within the oocyte during microinjection. Reducing invagination to minimize the disruption of the cytoplasm should be beneficial for the oocyte. It was hypothesized in this study that the microICSI device provided more support around the zona pellucida and oocyte during ICSI, reducing the invagination of the oolemma. However, porcine oocyte invagination scores were equivalent between C-ICSI and microICSI (Fig. 5d). This may reflect differences in membrane fluidity between species, as invagination levels are much higher in human oocytes than observed in porcine [28]. In addition, the type of oocyte maturation may have influenced invagination as the porcine oocytes were matured *in vitro* compared to *in vivo*-derived human oocytes. Further investigation will determine whether microICSI is able to reduce invagination in human oocytes.

High levels of parthenogenic cleavage were observed in both microICSI sham injected and non-injected control porcine oocytes (Fig. 6c), a previously reported phenomenon [31, 35]. The higher levels of cleavage observed here is likely attributable to evaluating cleavage at 96 hours rather than 48 h [31], allowing more opportunity for abnormal cleavage to occur. Additionally, fragmentation of the sham group tended to be higher than the sperm injected groups, and the non-injected controls showed significantly higher fragmentation than all other groups (Fig. 6a), suggesting that some level of injection-stimulated activation reduces membrane destabilization after four days of culture. Significantly, both the sham injected group and controls produced no blastocysts (Fig. 6d), a characteristic also reported in other studies of very low or absent blastocyst development [31, 35]. This indicates the blastocysts from C-ICSI and microICSI were a result of sperm induced oocyte activation. Assessment of cell numbers at the blastocyst stage revealed no difference between microICSI and C-ICSI groups. The overall blastocyst cell numbers are similar to those reported previously [31].

The microICSI device provided procedural benefits over conventional practice. This study demonstrated that microICSI improved embryologist workflow and operational time by reducing the amount of hand adjustments required for each injection and reducing injection time. C-ICSI requires the operator to efficiently control multiple devices including micromanipulators, the hydraulic air and/or oil pressure controllers, microscope stage, and microscope focus. When performing microICSI, an operator only needs to focus on a single spermatozoon in the injection pipette with one controller and micromanipulator. This reduces the operator attention required during polar body orientation, which is recommended to prevent meiotic spindle disruption during injection [10].

Loading oocytes into numbered microwells of the microICSI array greatly improves traceability for systematic injections. Improved traceability for oocyte microinjection was also described in a previous publication, utilizing an earlier 2PP-printed device to inject fluorescent beads into mouse oocytes [25]. However, the addition of numbering to each microICSI well further improves the traceability, thereby improving quality control of the ICSI process and decreasing the opportunities for operator error. Under C-ICSI conditions, such risks are greater because of the less stringent traceability that accompanies moving oocytes from drop-to-drop. Furthermore, microICSI simplified the ICSI procedure by retaining oocytes within microwells, therefore requiring less oocyte locating and handling. During unloading, the base numbers are visible under a dissecting microscope and can be correlated with the embryologist’s workflow records of any immature oocytes or unsuccessful injections. However, the wells that provide this improved traceability increased microICSI oocyte handling time, with average loading and unloading time per 10 oocytes being 15 and 20 s longer respectively (Fig. 2 c and d). This time is recovered in the porcine model as mean individual microICSI oocyte injection time is 11 s faster than C-ICSI, including polar body orientation (Fig. 3d), saving nearly 2 min when injecting 10 oocytes.

There are several limitations of this study that should be considered when assessing the data. First, this study primarily involved porcine IVM-derived oocytes rather than controlled ovarian stimulated mature human oocytes. Lipid content in porcine oocytes make them opaque, increasing the difficulty of visualizing sperm during injection to ensure injection has been successful. There are also many possible workflow differences between the C-ICSI conducted in this study and other laboratory’s clinical practices that may influence the amount of benefit to human ICSI. This will be investigated in follow up studies. Another limitation is the use of a commercially available ICSI dish rather than one designed specifically to hold the 2PP part. This meant the oocytes sat well above the plane of the sperm drops on the bottom of the dish. Future designs will recess the part to enable the injector to move across from the sperm drop to the central plane of the oocyte for immediate injection without adjusting the injector height. Future studies will assess whole case timings as moving the injection pipette between the sperm drop and oocytes will be further optimized. A final limitation of microICSI is that adjustments to existing ICSI techniques are required to use it efficiently. Polar body orientation within microICSI requires smaller and shorter movements with the injection pipette rather than larger sweeping movements (Online Resource 7). However, with these adjustments, microICSI has been shown here as a device capable of supporting the complete ICSI procedure.

The improvements in procedural efficiency of porcine microICSI could have significant impact in human clinical laboratory practice, where the demand for ICSI and IVF is set to increase in the coming decades [1, 36]. Likewise, different workflows, dish setup, and user techniques may also improve C-ICSI efficiency. However, further studies may show there is reduced ICSI training time required for trainee embryologists to learn microICSI, which, combined with less set up time and procedure time, will provide flow-on impacts to alleviate the workload of skilled staff and mitigate the impact of the wider shortage of trained embryologists [1, 37]. Furthermore, a reduction in ICSI workload is likely to assist in reducing musculoskeletal disorders, which are the most prevalent occupational health issue impacting embryologists [38].

As no suction is required to hold the oocyte in the device, microICSI could readily be extended to other micromanipulation techniques currently requiring a holding pipette. For Robotic ICSI, the numbered array provides known locations of every oocyte to enable further automation of the ICSI process [15, 17, 39]. For Piezo ICSI, which reduces stress caused by the injector [13, 23], performing it with microICSI, which likely removes the stress caused by the holding pipette, could further improve ICSI outcomes by combining the benefits of these two techniques. Computational modelling, such as finite element analysis (FEA), could be used in future studies to analyse the forces on the oocyte during microinjection when using a holding pipette compared to a microICSI hemisphere [40]. Microinjection of beads into human oocytes within the microICSI device has demonstrated it may be transferable to clinical practice with similar reductions in time and complexity of the procedure. Further studies will examine the utility of microICSI in a clinical setting, evaluating the embryological outcomes as well as further procedural parameters.

In conclusion, this study demonstrated that microICSI has both developmental and procedural benefits over C-ICSI. Further refinement and testing for human oocyte microICSI could provide significant benefits to clinical outcomes, while reducing the time and skill burden of conducting the ICSI procedure to improve clinical workflow.

### Supplementary information

Online Resource 1 Further details on the ICSI dish setups. (PDF 514 kb)

Online Resource 2 Ideal example of polar body orientation and successful oocyte injection; video shows the workflow of polar body orientation and injection for a single porcine oocyte with matching hand adjustment camera footage compared between C-ICSI and microICSI. Individual timers are shown for C-ICSI and microICSI, counters indicate hand adjustments between controllers, 10x objective, 2x magnifier, Scale bars = 100 μm. (MP4 2211 kb)

Online Resource 3 Mean polar body orientation time and successful oocyte injection; video shows the workflow of polar body orientation and injection for a single porcine oocyte with matching hand adjustment camera footage compared between C-ICSI and microICSI. Individual timers are shown for C-ICSI and microICSI, counters indicate hand adjustments between controllers, 10x objective, 2x magnifier, Scale bars = 100 μm. (MP4 3167 kb)

Online Resource 4 Microinjection of a bead into a human metaphase II oocyte; video shows microinjection of a single human oocyte for conventional injection compared to injection within the microICSI device. Individual timers are shown for each technique, counters indicate hand adjustments between controllers, 10x objective, 2x magnifier, Scale bars = 100 μm. (MP4 3590 kb)

Online Resource 5 Further data on polar body orientation. (PDF 122 kb)

Online Resource 6 Further data on the impact of operator and ICSI rig on ICSI performance. (PDF 154 kb)

Online Resource 7 Polar body orientation of 10 porcine oocytes within the microICSI device; video shows porcine oocytes located in microwells 1-10, 10x objective, 2x magnifier, Scale bar = 250 μm. (MP4 11048 kb)

## Data Availability

Restrictions apply to the availability of the supporting data of this study. Upon reasonable request to the corresponding authors, and with permission of Fertilis Pty Ltd, supporting data can be provided.
