# Supplementary material for: A micro-fabricated device (microICSI) improves porcine blastocyst development and procedural efficiency for both porcine intracytoplasmic sperm injection and human microinjection
Source: J Assist Reprod Genet. 2024 Jan 18;41(2):297–309. doi: 10.1007/s10815-023-03018-0 (PMC10894805; doi:10.1007/s10815-023-03018-0)
Supplement: Supplementary file 1 — Further details on the ICSI dish setups. (PDF 514 kb) [file 10815_2023_3018_MOESM1_ESM.pdf]

Online Resource 1: Further details on the ICSI dish setups

Manuscript Title: A micro-fabricated device (microICSI) improves porcine blastocyst development and procedural efficiency for both porcine intracytoplasmic sperm injection and human microinjection.

Journal: Journal of Assisted Reproduction and Genetics

Authors:
Hanna J. McLennan[1], Shauna L. Heinrich[1], Megan P. Inge[1], Samuel J. Wallace[2], Adam J. Blanch[1], Llewelyn Hails[1], John P. O'Connor[1], Michael B. Waite[1], Stephen McIlfatrick[3, 4], Mark B. Nottle[3, 4], Kylie R. Dunning[3, 4, 5, 6], David K. Gardner[1, 7, 8], Jeremy. G. Thompson[1, 4, 9], Allison K. Love[1].

Affiliations:
[1]Fertilis Pty Ltd, Frome Road, Helen Mayo South, The University of Adelaide, Adelaide, SA, 5005, Australia
[2]Virtual Ark Pty Ltd, 73 Woolnough Road, Semaphore, SA, 5019, Australia
[3]School of Biomedicine, Faculty of Health and Medical Sciences, The University of Adelaide, Adelaide, SA, 5005, Australia
[4]Robinson Research Institute, Adelaide Medical School, The University of Adelaide, Adelaide, SA, 5005, Australia
[5]Australian Research Council Centre of Excellence for Nanoscale BioPhotonics, The University of Adelaide, Adelaide, SA, 5005, Australia
[6]Institute for Photonics and Advanced Sensing, The University of Adelaide, Adelaide, SA, 5005, Australia
[7]Melbourne IVF, East Melbourne, VIC, 3002, Australia
[8]School of BioSciences, University of Melbourne, Parkville, VIC, 3010, Australia
[9]ART Lab Solutions Pty Ltd, 10 Pulteney Street, Adelaide, SA, 5005, Australia

Corresponding author(s):
H. J. McLennan (hanna.mclennan@fertil.is) and J. G. Thompson (jeremy@fertil.is)

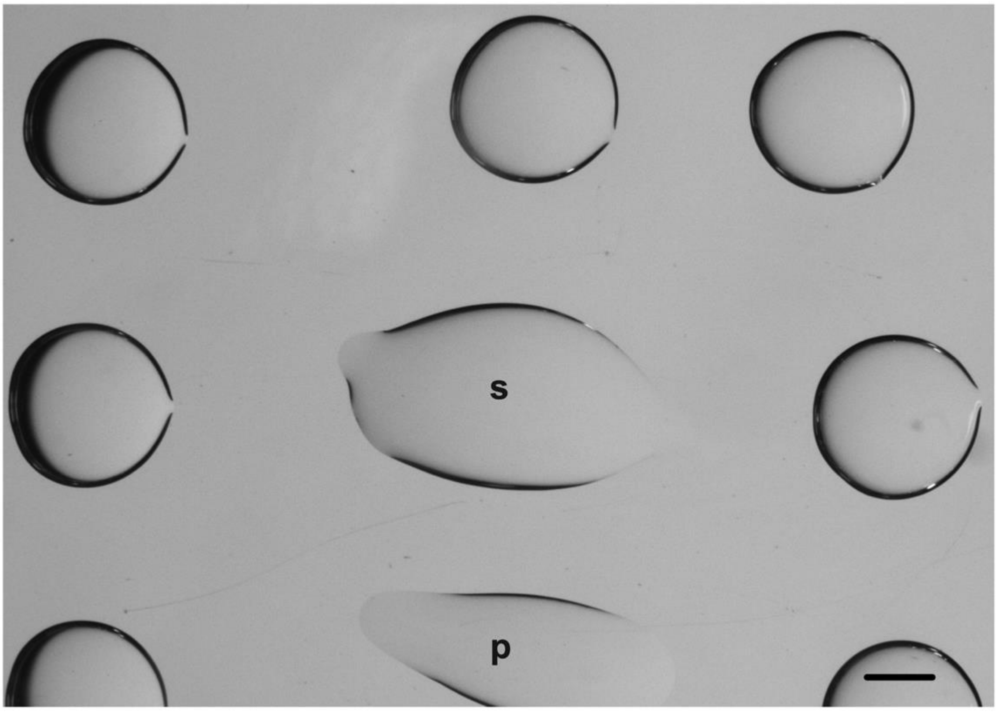

Fig. O1a: Conventional ICSI (C-ICSI) Dish Layout (Scale bar = 1mm); seven 5 μL media droplets, one 5 μL 7% PVP droplet indicated by '**p**', and one 10 μL 3.5% PVP droplet indicated by '**s**' for the sperm arranged in a 3x3 grid in the centre of a Vitrolife ICSI dish and covered in 5 mL of paraffin oil.

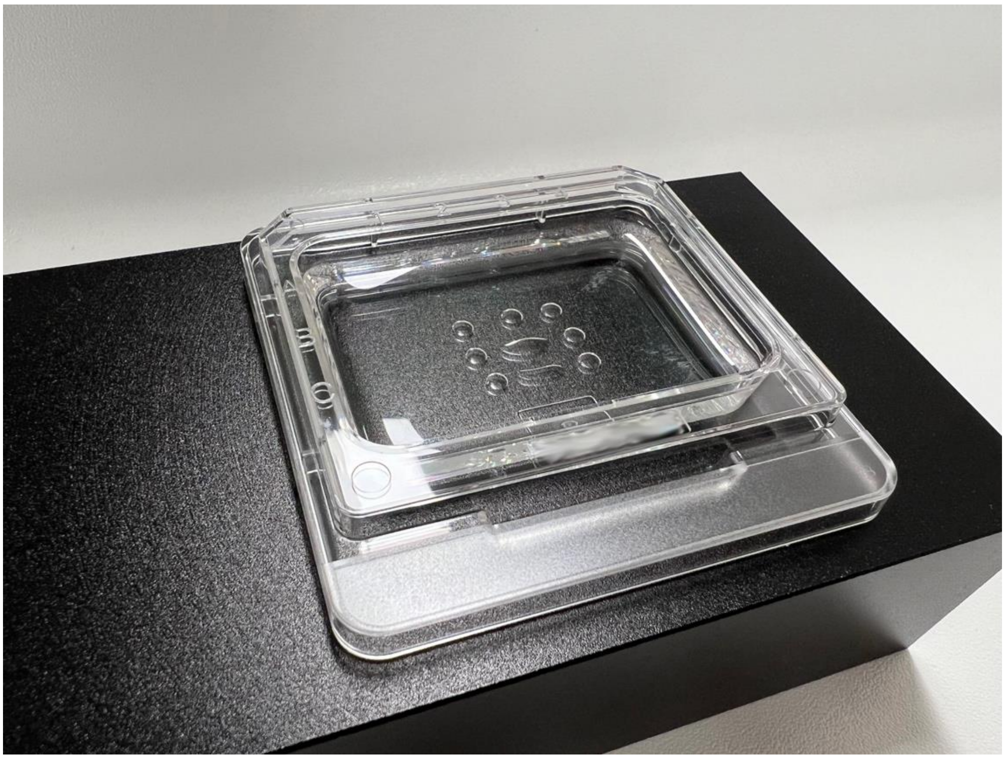

Fig. O1b: Photograph of a prepared C-ICSI dish. Media droplets are in the centre of the dish and covered by 5 mL of paraffin oil.

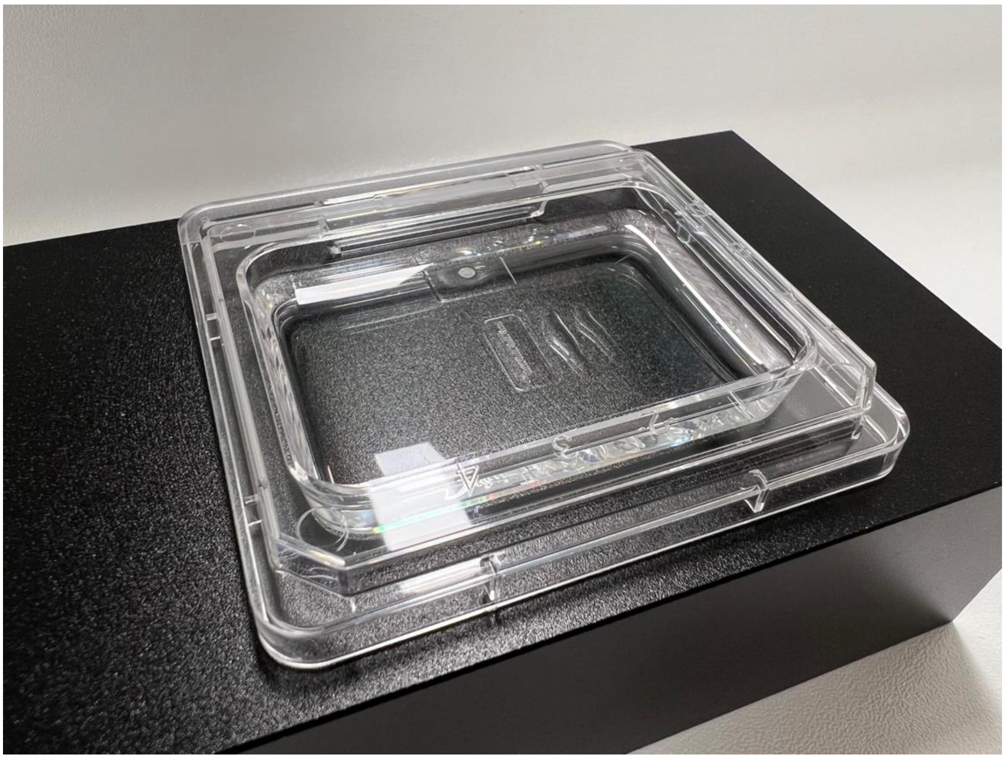

Fig. O1c: Photograph of a prepared microICSI dish. The microICSI array is covered by 20 μL of media with PVP strips to the right of the array, which is all covered by 5 mL of paraffin oil.
