# Supplementary material for: A micro-fabricated device (microICSI) improves porcine blastocyst development and procedural efficiency for both porcine intracytoplasmic sperm injection and human microinjection
Source: J Assist Reprod Genet. 2024 Jan 18;41(2):297–309. doi: 10.1007/s10815-023-03018-0 (PMC10894805; doi:10.1007/s10815-023-03018-0)
Supplement: Supplementary file 5 — Further data on polar body orientation. (PDF 122 kb) [file 10815_2023_3018_MOESM5_ESM.pdf]

Online Resource 5: Further data on polar body orientation

Manuscript Title: A micro-fabricated device (microICSI) improves porcine blastocyst development and procedural efficiency for both porcine intracytoplasmic sperm injection and human microinjection.

Journal: Journal of Assisted Reproduction and Genetics

Authors:
Hanna J. McLennan[1], Shauna L. Heinrich[1], Megan P. Inge[1], Samuel J. Wallace[2], Adam J. Blanch[1], Llewelyn Hails[1], John P. O'Connor[1], Michael B. Waite[1], Stephen McIlfatrick[3, 4], Mark B. Nottle[3, 4], Kylie R. Dunning[3, 4, 5, 6], David K. Gardner[1, 7, 8], Jeremy. G. Thompson[1, 4, 9], Allison K. Love[1].

Affiliations:
[1]Fertilis Pty Ltd, Frome Road, Helen Mayo South, The University of Adelaide, Adelaide, SA, 5005, Australia
[2]Virtual Ark Pty Ltd, 73 Woolnough Road, Semaphore, SA, 5019, Australia
[3]School of Biomedicine, Faculty of Health and Medical Sciences, The University of Adelaide, Adelaide, SA, 5005, Australia
[4]Robinson Research Institute, Adelaide Medical School, The University of Adelaide, Adelaide, SA, 5005, Australia
[5]Australian Research Council Centre of Excellence for Nanoscale BioPhotonics, The University of Adelaide, Adelaide, SA, 5005, Australia
[6]Institute for Photonics and Advanced Sensing, The University of Adelaide, Adelaide, SA, 5005, Australia
[7]Melbourne IVF, East Melbourne, VIC, 3002, Australia
[8]School of BioSciences, University of Melbourne, Parkville, VIC, 3010, Australia
[9]ART Lab Solutions Pty Ltd, 10 Pulteney Street, Adelaide, SA, 5005, Australia

Corresponding author(s):
H. J. McLennan (hanna.mclennan@fertil.is) and J. G. Thompson (jeremy@fertil.is)

Tab. O5a: Comparison of polar body orientation time and the number of pipette touches required to orient a single porcine oocyte between C-ICSI and microICSI showing the number of oocytes analysed (two-tailed unequal variance t-test)**.**

	Operator	C-ICSI	microICSI	p-value
Oocyte Count	1	50	58	-
	2	55	46	-
Timing (secs)	1	8.74	11.41	0.059
	2	11.24	11:65	0.837
Pipette Touches	1	3.8	5.8	0.001
	2	4.2	6.2	0.022

Polar body orientation time was calculated over three replicates for C-ICSI and microICSI by reviewing high frame rate video footage captured from the microscope camera. The start point of the orientation was defined as 1 to 2 frames before the injector needle touched the oocyte, and the end point was defined as 1 to 2 frames after the injector needle last touched the oocyte when the polar body was in either the 12 or 6 o'clock position. For C-ICSI, some oocyte orientations did not require the injector needle to touch the oocyte, the polar body was already in the 12 or 6 o'clock position. In those cases, the timing was based on how long the oocyte took from when it first moved due to the suction from the holding pipette, until when it was in a fixed position. For oocytes where there was no visible polar body after orientation was attempted, those data were excluded. For oocytes where there were significant cumulus cells impeding orientation, those data were also excluded.

In the porcine model, polar body orientation involves a searching phase and an orientation phase, due to the opaque nature of the cytoplasm. In the searching phase, the operator is performing rotations along two axes to visualise where the polar body may sit. Once it has been identified, the operator is performing deliberate operations to bring it to the 12 or 6 o'clock position. In the human model, due to the transparent nature of the cytoplasm, there is no searching phase required.
