# Supplementary material for: A micro-fabricated device (microICSI) improves porcine blastocyst development and procedural efficiency for both porcine intracytoplasmic sperm injection and human microinjection
Source: J Assist Reprod Genet. 2024 Jan 18;41(2):297–309. doi: 10.1007/s10815-023-03018-0 (PMC10894805; doi:10.1007/s10815-023-03018-0)
Supplement: Supplementary file 6 — Further data on the impact of operator and ICSI rig on ICSI performance. (PDF 154 kb) [file 10815_2023_3018_MOESM6_ESM.pdf]

Online Resource 6: Further data on the impact of operator and ICSI rig on ICSI performance

Manuscript Title: A micro-fabricated device (microICSI) improves porcine blastocyst development and procedural efficiency for both porcine intracytoplasmic sperm injection and human microinjection.

Journal: Journal of Assisted Reproduction and Genetics

Authors:
Hanna J. McLennan[1], Shauna L. Heinrich[1], Megan P. Inge[1], Samuel J. Wallace[2], Adam J. Blanch[1], Llewelyn Hails[1], John P. O'Connor[1], Michael B. Waite[1], Stephen McIlfatrick[3, 4], Mark B. Nottle[3, 4], Kylie R. Dunning[3, 4, 5, 6], David K. Gardner[1, 7, 8], Jeremy. G. Thompson[1, 4, 9], Allison K. Love[1].

Affiliations:
[1]Fertilis Pty Ltd, Frome Road, Helen Mayo South, The University of Adelaide, Adelaide, SA, 5005, Australia
[2]Virtual Ark Pty Ltd, 73 Woolnough Road, Semaphore, SA, 5019, Australia
[3]School of Biomedicine, Faculty of Health and Medical Sciences, The University of Adelaide, Adelaide, SA, 5005, Australia
[4]Robinson Research Institute, Adelaide Medical School, The University of Adelaide, Adelaide, SA, 5005, Australia
[5]Australian Research Council Centre of Excellence for Nanoscale BioPhotonics, The University of Adelaide, Adelaide, SA, 5005, Australia
[6]Institute for Photonics and Advanced Sensing, The University of Adelaide, Adelaide, SA, 5005, Australia
[7]Melbourne IVF, East Melbourne, VIC, 3002, Australia
[8]School of BioSciences, University of Melbourne, Parkville, VIC, 3010, Australia
[9]ART Lab Solutions Pty Ltd, 10 Pulteney Street, Adelaide, SA, 5005, Australia

Corresponding author(s):
H. J. McLennan (hanna.mclennan@fertil.is) and J. G. Thompson (jeremy@fertil.is)

In this study, ICSI was performed on two rigs, setup as described in the methodology, to minimise the aging of the oocytes post *in vitro* maturation (IVM). The two operators had varying levels of experience with both C-ICSI and microICSI prior to the experiment commencing. The first operator had 20 years of ICSI experience across multiple laboratories and had accumulated 35 hours of microICSI practice prior to experimentation. The second operator had completed 22 hours of C-ICSI training with an additional 5 hours of microICSI training prior to experimentation. The data below separate injection time and number of hand adjustments by operator and rig.

Tab. O6a: Comparison of injection time and hand movements required across each ICSI rig (two-tailed unequal variance t-test).

	Treatment	ICSI Rig 1	ICSI Rig 2	p-value
Oocyte Count	C-ICSI	251	420	-
	microICSI	313	339	-
Injection Time (secs)	C-ICSI	46.82	45.02	1.1E-01
	microICSI	15.00	17.21	3.9E-06
Right Hand Movements	C-ICSI	2.9	3.4	3.4E-06
	microICSI	1.7	2.1	6.3E-20
Left Hand Movements	C-ICSI	6.1	6.7	1.1E-03
	microICSI	1.1	1.1	1.0E-01

Tab. O6b: Comparison of injection time and hand movements required for each operator (two-tailed unequal variance t-test).

	Treatment	Operator 1	Operator 2	p-value
Oocyte Count	C-ICSI	316	355	-
	microICSI	313	339	-
Injection Time (secs)	C-ICSI	47.40	44.18	3.3E-03
	microICSI	15.00	17.21	3.9E-06
Right Hand Movements	C-ICSI	3.0	3.4	2.2E-05
	microICSI	1.7	2.1	6.3E-20
Left Hand Movements	C-ICSI	6.1	6.7	1.4E-04
	microICSI	1.1	1.1	1.0E-01

The data collected has a tight coupling between ICSI Rig and operator, with Operator 1 working predominantly on ICSI Rig 1, and Operator 2 predominantly on ICSI Rig 2. The differences in the timing data are likely due to several factors. The reaction times of the time recorders, ICSI Rig holding pipette raising and lowering speed and the differences in the self-reported start and stop points of the injections. ICSI Rig 1 required the operator to hold a button to raise or lower the holding pipette into focus, while ICSI Rig 2 only required the operator to press a button to shift between two preset Z heights. Data was excluded if an individual injection did not contain a polar body, or if the capture of hand movements was compromised or incomplete. As the largest difference in timing is 3 seconds, which is largely attributable to the differences in data capture specified above, the operator and rig used

would have minimally impacted the results reported in the main manuscript. Similarly, the slight difference in hand movements between rigs was due to the difference in microscope controls between the two rigs.
